# Compound heterozygous mutations in electron transfer flavoprotein dehydrogenase identified in a young Chinese woman with late-onset glutaric aciduria type II

**DOI:** 10.1186/s12944-017-0576-5

**Published:** 2017-09-26

**Authors:** Ying Xue, Yun Zhou, Keqin Zhang, Ling Li, Abudurexiti Kayoumu, Liye Chen, Yuhui Wang, Zhiqiang Lu

**Affiliations:** 1 Department of Endocrinology, Tongji Hospital of Tongji University, Tongji University School of Medicine, Shanghai, 200065 China; 2 Institute of Cardiovascular Science, Peking University and Key laboratory of Molecular Cardiovascular Science, Ministry of Education, Beijing, 100191 China; 3 Department of Endocrinology, Zhongshan Hospital, Fudan University, Shanghai, 200032 China

**Keywords:** Glutaric aciduria type II (GA II), Electron transfer flavoprotein dehydrogenase (*ETFDH)*, Autosomal recessive disorder

## Abstract

**Background:**

Glutaric aciduria type II (GA II) is an autosomal recessive disorder affecting fatty acid and amino acid metabolism. The late-onset form of GA II disorder is almost exclusively associated with mutations in the electron transfer flavoprotein dehydrogenase (*ETFDH*) gene. Till now, the clinical features of late-onset GA II vary widely and pose a great challenge for diagnosis. The aim of the current study is to characterize the clinical phenotypes and genetic basis of a late-onset GAII patient.

**Methods:**

In this study, we described the clinical and biochemical manifestations of a 23-year-old female Chinese patient with late-onset GA II, and performed genomic DNA-based PCR amplifications and sequence analysis of *ETFDH* gene of the whole pedigree. We also used in-silicon tools to analyze the mutation and evaluated the pathogenicity of the mutation according to the criteria proposed by American College of Medical Genetics and Genomics (ACMG).

**Results:**

The muscle biopsy of this patient revealed lipid storage myopathy. Blood biochemical test and urine organic acid analyses were consistent with GA II. Direct sequence analysis of the *ETFDH* gene (NM_004453) revealed compound heterozygous mutations: c.250G > A (p.A84T) on exon 3 and c.920C > G (p.S307C) on exon 8. Both mutations were classified as “pathogenic” according to ACMG criteria.

**Conclusions:**

In conclusion, our study described the phenotype and genotype of a late-onset GA II patient, reiterating the importance of *ETFDH* gene screening in these patients.

**Electronic supplementary material:**

The online version of this article (10.1186/s12944-017-0576-5) contains supplementary material, which is available to authorized users.

## Background

Glutaric aciduria type II (GA II), also known as multiple Acyl-CoA dehydrogenase deficiency (MADD), is a rare autosomal recessive disorder of fatty acid, amino acid and choline metabolism caused by a defect in the alpha or beta subunit of the mitochondrial electron transfer flavoprotein (ETFA, ETFB) protein or the electron transfer flavoprotein dehydrogenase (ETFDH) protein [1]. The clinical manifestation of GA II is heterogeneous, from severe neonatal form to mild late-onset form. Children with the severe form usually show a fatal course in the neonatal period. In the mild form, the age of onset and the symptoms are variable with intermittent episodes, challenging the clinical diagnosis [2]. These patients often present with fluctuating proximal muscle weakness or episodes of rhabdomyolysis. Other manifestations include sensory neuropathy, loss of tendon reflex, fatty liver, etc. [2]. Current clinical diagnosis is mainly based on tandem mass spectrometry findings of elevated plasma levels of short, medium, and long chain acylcarnitines. However, in late-onset patients, the elevation of acylcarnitine levels may be mild and atypical, or detectable only during an acute episode. Under these circumstances, genetic screening is the key to establish a definitive diagnosis.

It has long been well-known that the mild form of GAII has exceptionally good response to riboflavin supplementation, although its true mechanism remains to be clarified. Recent work also suggests that most late-onset GA II patients with good response to riboflavin have mutations in the *ETFDH* gene. The *ETFDH* gene is located at 4q32.1, with a total of 13 exons. It encodes the electron transfer flavoprotein: ubiqionone oxidoreductase (ETF: QO) [3]. As a component in mitochondrial respiratory chain, ETF: QO forms a short pathway with ETF to transfer electrons from mitochondrial flavoprotein dehydrogenases to the ubiquinone pool [4].

So far, more than 190 mutations in the *ETFDH* gene, including point mutations, nonsense mutations, insertions, deletions, splicing mutations, have been reported according to the Human Gene Mutation Database (HGMD) [5]. The genotype and phenotype in *ETFDH*-mutant GAII patients were previously reported to be correlated. Nonsense mutations leading to truncation or missense mutations affecting protein stability in both alleles were generally associated with a severe phenotype [6, 7]. However, according to a recent cohort study, in the late-onset mild forms, the correlation between genotype and phenotype (including age of onset, disease severity and response to treatment) is not well-established [8].

Herein, we describe the clinical presentation and course of a 23-year-old female who was diagnosed as late-onset GA II. We identified heterozygous mutations in *ETFDH* gene, including a p.A84T, located at the flavin adenine dinucleotide (FAD)-binding domain and a p.S307C mutation, located within the ubiquinone (UQ)-binding domain of ETF dehydrogenase.

## Methods

### Case description

A 23-year-old female was admitted to our hospital because of exercise intolerance and general muscle weakness, especially in her lower limbs, for 3 months. The symptom of weakness in her neck and proximal limb muscles was noted to have aggravated in last 1 month. She had difficulty walking long distances, gradually she was unable to raise her head and arms away from bed. Two weeks before admission, she experienced intermittent vomiting and weight loss. Her prenatal history and early development were both normal, and her life history was unremarkable. Physical and neurological examinations showed neck and proximal muscle weakness (manual muscle testing (MMT) score 3/5 in neck, 2/5 in lower limbs, 4/5 in upper arm).

Besides her clinical history, other clinical details including blood biochemical examinations, blood acylcarnitine analysis, urine organic acids spectrum, muscle magnetic resonance imaging (MRI), CT scan of the abdomen, electrocardiogram (EEG) were also collected. Muscle biopsy was performed on the right biceps brachii and immunohistochemistry was performed using frozen sections. The patient was treated with riboflavin (120 mg/day) and carnitine supplements (1 g/day). Two days after riboflavin and carnitine treatment, she experienced a severe respiratory infection and developed rapidly progressive quadriparesis with acute respiratory failure. Despite immediate tracheal intubation, the patient succumbed to sudden heart arrest, which showed no response to chest compressions, intravenous adrenalin, or electric defibrillation.

The proband and her family were Han Chinese and lived in southern China. Her parents were not consanguineous and had no myopathic symptom. The younger brother of the proband, a 15-year old boy, had no history of neuromuscular disease. After written informed consent was obtained, blood samples of all family members were collected for genetic testing.

### Mutation analysis

Genomic DNA-based PCR amplifications and sequence analysis of *ETFDH* gene of the whole pedigree was performed. Briefly, genomic DNA from peripheral blood leukocytes was isolated by proteinase K digestion and phenol/chloroform extraction. All of the exons and exon-intron boundaries of the *ETFDH* (GenBank NM_004453) gene were amplified by PCR with high fidelity KOD-plus polymerase from the hyperthermophilic Archaeon *Thermococcus kodakaraensis* KOD1 (TOYOBO, Japan) using primers listed in Table 1. The amplified PCR products were purified from agarose gel using QIAquick Gel Extraction Kit (Qiagen, Hilden, Germany) and sequenced via the ABI3730XL sequencer (Applied Biosystems, CA, USA). Mutations were confirmed using control DNA from 200 unaffected Chinese individuals after obtaining a written informed consent.

**Table 1:** Primers and amplicons for detecting *ETFDH* gene mutation

Name	Sequence of primers	Amplicon(bp)
ETFDH-Exon1-F	CAATCGATCTCGAAGGGCACT	659 bp
ETFDH--Exon1-R	TTAATTCTACCAACTGGGGCAAC	
ETFDH--Exon2-F	GTTAAGCCTGACATGAGCTAAATTG	537 bp
ETFDH--Exon2-R	TAATTGTCGTGTTGTTGACTGAAGG	
ETFDH--Exon3-F	TGTGCAAAACACAGGGAGAATTTC	512 bp
ETFDH--Exon3-R	AGCCTGGGCAACAAGAGTGAAA	
ETFDH--Exon4-F	AGGAGAAACACTTGAACCCAGGA	502 bp
ETFDH--Exon4-R	AGTAAGTCCTTCAAATATCTGGGTCTC	
ETFDH--Exon5-F	TGAAAGTGTGACCATCAATGTAGCA	437 bp
ETFDH--Exon5-R	TTGATGGGGGTACAACATGAGGA	
ETFDH--Exon6-F	CTTTCTCATCAAGGTTGTGGCAT	405 bp
ETFDH--Exon6-R	TTGGAGAGATGGGGTTTCACTTT	
ETFDH--Exon7-F	CCATTGGCAGAGGAGCTGGAT	683 bp
ETFDH--Exon7-R	CAACACTTGATACGACAATTCCAAC	
ETFDH--Exon8-F	CACCGTGCCCAGCCTTCTT	477 bp
ETFDH--Exon8-R	AATGACACAGCAGCCATAAGCACT	
ETFDH--Exon9-F	CCAGAGCACAAGGATTTCTTAATATG	693 bp
ETFDH--Exon9-R	ATTTGTTGTAGAGATGGGGTTTCG	
ETFDH—Exon10-F	ATTTTTCAGCCTTTCCCTACAGC	551 bp
ETFDH—Exon10-R	GCCTACCAAAGTGCTGGGATTAC	
ETFDH—Exon11-F	CCAACCTGGGTGACAGAGCAA	580 bp
ETFDH—Exon11-R	CTTCAACAATTTTGAGGGAAATGCT	
ETFDH—Exon12-F	TGAGGGCTAGTCATATTTCTTTGGT	444 bp
ETFDH—Exon12-R	TTTCTAAGGAATGGAAGGAGATACAG	
ETFDH—Exon13-F	TGAGAGGATGACTGTGAATAAGGGA	689 bp
ETFDH—Exon13-R	GAACTGAAGAGGTAGGAAGATGCTG	

### Bioinformatic analysis

Two in silicon tools were used for the analysis of mutation pathogenicity. The machine learning method predicts variants according to Bayesian methods (PolyPhen2) [9], or mathematical operations (SIFT) [10]. Multiple sequence alignment was performed using ClustalX [11]. The X-ray crystal structure of pig ETF:QO (Protein Data Bank entry 2GMH, resolution 2.5 Å) [12] was used as the template to construct human wild type and S307C mutational *ETFDH* structure models. The sequence of human *ETFDH* was retrieved from UniProt (http://www.uniprot.org/uniprot/Q16134). The 3D models of human *ETFDH* complexed with substrates (ubiquinone, FAD, and 4Fe4S) were generated using MODELLER (version 9.12) [13].

## Results

### Clinical information

Laboratory examinations revealed an elevated plasma creatine kinase (CK) level up to 26,997 IU/ml (normal 26-140 U/L). Other dramatically abnormal parameters included: alanine aminotransferase (ALT) 598 U/L (normal 9-50 U/L), aspartate aminotransferase (AST) 5029 U/L (normal 15-40 U/L), creatine kinase-MB (CKMB) 944 U/L (normal 0-23 U/L), myoglobin >3000 ng/mL (28-72.0 ng/mL), lactate dehydrogenase (LDH) >4000 U/L (109-245 U/L).

Organic acid analysis of the urine sample showed a marked increase in a variety of dicarboxylic acids (Table 2), including glutarate, adipate, 2-hydroxyglutaric acid, 3-hydroxyglutaric acid, 2-hydroxy adipic acid, orotic acid, 4-hydroxy-phenyllactic acid, and ethylmalonic acid. The organic acid profile in urine was highly suggestive of GA II. Acylcarnitine analysis of blood sample was unremarkable. No elevation of short (C6), medium (C8, C10:1, C10) or long chain (C14:1, C14) acylcarnitines was observed.

**Table 2:** GC / MS analysis of the organic acid of the urine sample

No.	Test item	Results	Normal average	Normal min	Normal max	Results (Average)	Results (Max)
1	glutarate	105.21	1.9	0	4	55.37	26.3
2	adipic acid	58.55	3	0.5	5	19.52	11.71
3	2 - hydroxy glutaric acid	143.58	2.3	0.6	5.9	62.42	24.34
4	2-hydroxy adipic acid	43.54	1.2	0	2	36.28	21.77
5	orotic acid	35.2	0.3	0	1.5	117.33	23.12
6	isovaleryl-glycine	3.1	0.1	0	0.4	31.02	7.75
7	4-hydroxy phenyllactic acid	255.05	1.8	0	7	141.7	36.44
8	ethylmalonic acid	10.03	0.9	0	5.2	11.15	1.62
9	4 - hydroxy benzene pyruvic acid	4.09	0.2	0	0.9	20.46	4.54

**Fig. 1:**
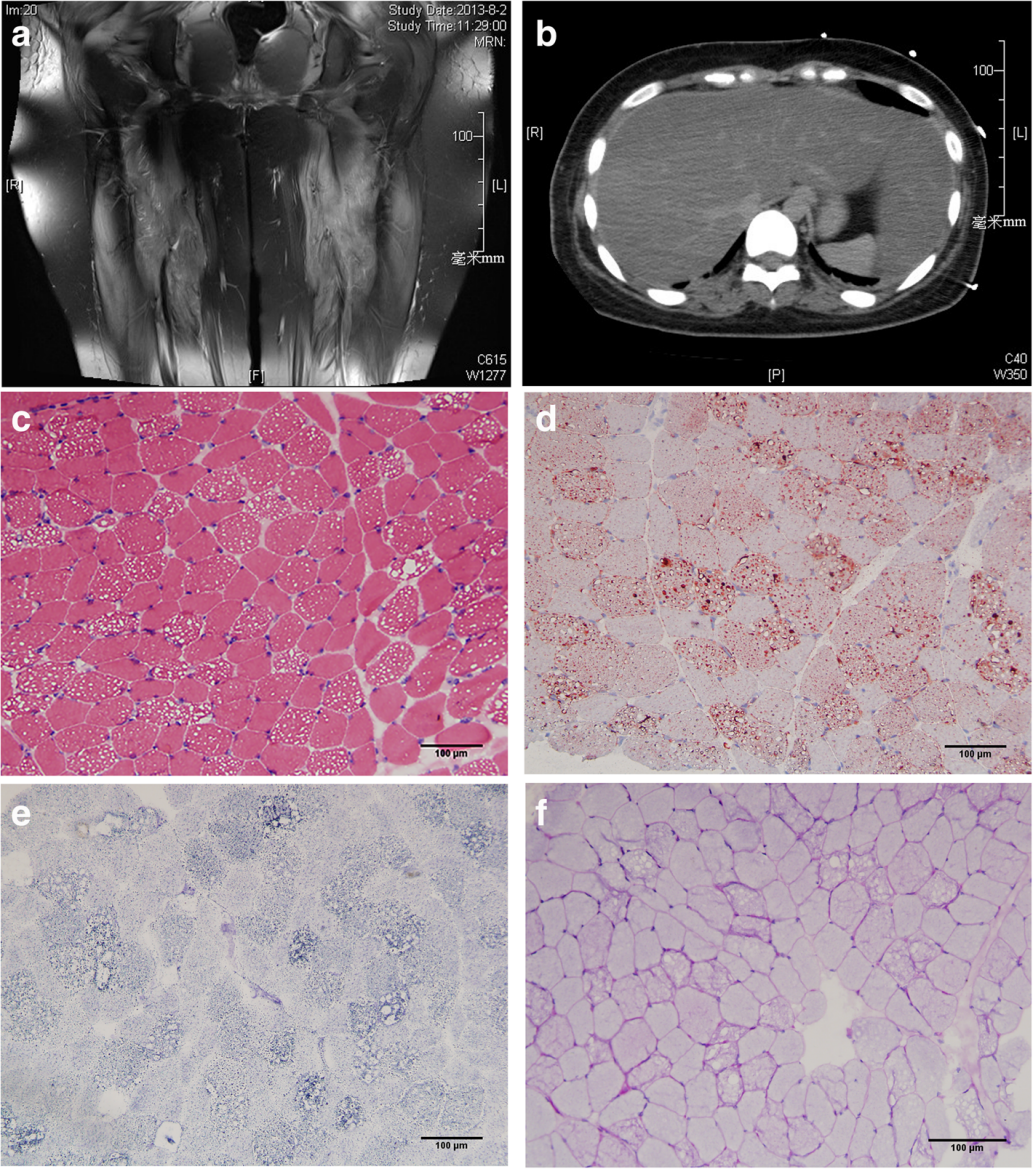
Radiologic and histologic findings of the proband. **a** Diffuse muscle injury in the lower limbs of the proband as revealed by high signal intensity on T2-weighted MR imaging. **b** Significantly lower density of the liver compared with that of the spleen on abdominal CT scanning. **c** H&E staining showed vacuolar myopathy, with fine vacuoles found mainly in type I fibers. **d** The Oil Red O staining showed diffuse lipid droplets accumulation, predominantly in type I fibers. **e** NADH staining indicated disorganized myofibrillar network. **f** PAS staining did not show excessive glycogen content. (C ~ F) Original magnification ×200

Muscle MRI of lower limbs showed high signal intensity areas in the bilateral lower limb muscles on T2-weighted MR imaging, indicating diffuse muscle injury (Fig. 1a). CT scans of the abdomen indicated severe lipid accumulation in the liver (fatty liver) as suggested by significantly lower density compared with that of the spleen (Fig. 1b).

Histochemical staining of muscle frozen sections revealed vacuolar myopathy, with numerous fine vacuoles found mainly in type 1 fibers (Fig. 1c). The Oil Red O stain showed diffuse lipid droplets accumulation, predominantly in type 1 fibers (Fig. 1d). Nicotinamide adenine dinucleotide (NADH) staining indicated the myofibrillar network was disorganized in type 1 fibers, and no excessive glycogen content was observed on periodic acid-schiff (PAS) staining (Fig. 1f and g). The pathological findings were compatible with lipid storage myopathy. Histological findings of a control case were shown in Additional file 1: Figure S1.

### Molecular studies

**Fig. 2:**
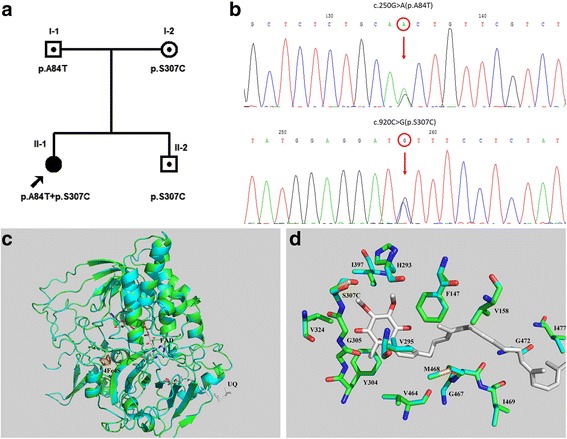
Identification of the *ETFDH* gene mutations and the ETFDH protein structural analysis. **a** Pedigree of the family based on direct sequencing of the *ETFDH* gene. **b** Electropherogram of the proband. c.250G > A (p.A84T) mutation at exon 3 and c.920C > G (p.S307C) mutation at exon 8 of the *ETFDH* gene were identified. **c** The 3D models of human ETFDH structures. **d** The wild type and S307C mutational structures showed cyan and green cartoons. Oxygen atoms are shown in red and nitrogen atoms in blue. Carbon atoms of wild type, S307C mutation and ubiquinone are shown in cyan, green and white, respectively. The phosphorus atom of mutational Cys307 is shown in yellow-orange. Crucial residues in the binding site of ubiquinone are shown as stick and labeled. Figures were generated by PyMol

Direct sequencing of all 13 exons of *ETFDH* gene revealed compound heterozygous mutations in the proband: c.250G > A (p.A84T) on exon 3 and c.920C > G (p.S307C) on exon 8 (Fig. 2b). Her father carried a c.250G > A (p.A84T) on exon 3, while her mother carried a c.920C > G (p.S307C) on exon 8. Her younger brother, a 17-year old boy, inherited c.920C > G (p.S307C) from his mother. The missense mutation c.920C > G (p.S307C) was not identified in a screening of 200 control chromosomes in Chinese individuals. No mutation was identified in *ETFA* or *ETFB* gene.

### Bioinformatic and structural analysis

Both silicon tools, namely PolyPhen-2 and SIFT, supported that p.S307C of *ETFDH* was a deleterious mutation (Table 3). The human and pig *ETFDH* proteins share 95% sequence identity, which makes homology modeling very reasonable. The results showed a minimal difference between the wild type and mutant structures, even in the ubiquinone binding pocket for p.S307C mutation (Fig. 2d). It should be mentioned that, the structure of amino acid serine (S) was very close to cysteine (C). The only difference in the chemical structure was the Sulfur atom instead of the Oxygen atom.

**Table 3:** In silicon prediction of deleterious effect for p.S307C of *ETFDH*

Tool	Results	Interpretation
PolyPhen-2	Probably damaging with a score of 1.000	Deleterious
SIFT	Median information content (MIC): 2.96	Low confidence of deleterious mutation with MIC above 3.25

## Discussion

GA II is mainly caused by homozygous or compound heterozygous mutations in *ETFA, ETFB,* or *ETFDH* genes. Mutations in *ETFA* and *ETFB* are usually associated with the neonatal forms, whereas *ETFDH* mutations often present as late-onset forms. For the late-onset form, the clinical manifestation varies considerably. So far, most GA II patients were reported from East Asia, especially mainland China. A recent study reported the clinical features and *ETFDH* mutation spectrum of 90 unrelated patients pooled from two major centers in China. Several hot spots were described: c.250G > A (p.A84T), c.770A > G (p.Y257C) and c.1227A > C (p.L409F) with a frequency of 12.2%, 15.0% and 12.2% in Chinese, respectively [8].

According to American College of Medical Genetics and Genomics (ACMG), the criterion for classification of pathogenic or likely pathogenic variants is weighted as very strong (PVS1), strong (PS1–4), moderate (PM1–6), or supporting (PP1–5) [5]. Several independent studies have consistently reported that the c.250G > A (p.A84T) mutation was the most common mutation in GAII patients from Southern China, as well as those from Taiwan, Hong Kong, Thailand and Singapore (PS1) [14, 15]. Consistently, the current proband and her father, both carrying the c.250G > A (p.A84T) mutation, lived in Zhejiang province, geographically classified as Southern China. The p.A84T mutation was previously reported to cause reduced ETFDH protein expression in vivo (PS3) [16]. The prevalence of p.A84T in GAII patients was significantly increased compared with the prevalence in East Asians (PS4) [15]. The p.A84T mutation itself was a mutational hot spot and located in a critical and well-established functional domain (FAD domain) (PM1). Thus, it was classified as “pathogenic” according to the criteria proposed by ACMG (3PS + 1 PM) [5].

In contrast, the c.920C > G (p.S307C) mutation was relatively new. The p.S307C mutation was previously reported and associated with GA II (PS1) [17, 18]. The p.S307C mutation was located in a critical and well-established functional domain (UQ-binding domain) (PM1) [18]. Furthermore, this variant was neither found in Exome Aggregation Consortium (ExAC) nor 1000 Genomes Project (PM2). Taking co-segregation into account in the pedigree, the p.S307C mutation was in trans with a pathogenic variant p.A84T (PM3). The deleterious effect was supported by multiple lines of computational evidence such as PolyPhen2 and SIFT (PP3). The phenotype of the patient is highly consistent with the phenotype caused by the *ETHDH* gene (PP4). Based on these findings, it should also be classified as “pathogenic” according to the criteria proposed by ACMG(1PS + 3 PM + 2PP) [5].

The c.920C > G (p.S307C) mutation was previously described in only two patients. Both patients were Han Chinese, whose age of onset were much older than our patient [17, 18]. The features of these three patients carrying the p.S307C mutation were summarized in Additional file 2: Table S1. One patient initially manifested as Bent spine syndrome. Both patients had limb and lip numbness, and EMG showed no sensory nerve action potential, suggesting severe sensory neuropathy besides muscle weakness [17, 18]. However, in the current case, progressive muscle weakness was the major symptom, while sensory nerve action potential was preserved. CT scan of the abdomen in our patient indicated severe lipid accumulation in the liver. Some GA II patients were previously reported to have fatty liver [19, 20]. In one case, liver lipid accumulation relieved after riboflavin supplementation [20]. Since the patient died unexpectedly, we were unable to see an effect of riboflavin supplementation on fatty liver.

The ETF:QO protein acts in conjunction with ETF to govern electron transport from several flavoprotein acyl-CoA dehydrogenases to the main respiratory chain in mitochondria [12]. Residues A84 and S307 are located in the flavin adenine dinucleotide (FAD)-binding domain and the ubiquinone (UQ)-binding domain of ETF dehydrogenase, respectively. Both of the codons are highly evolutionarily conserved. Previous studies have suggested that A84T mutation could affect the interaction between FAD and ETF:QO, thus, could reduce the efficiency of electron transfer [21]. S307, together with Y304, G305 and G306, belong to the β-sheet 11, which wrap around the C5 methyl, O4 carbonyl, and C3 methoxy groups of UQ. In particular, the O4 atom of UQ makes hydrogen bonds to the backbone nitrogen of G306 and carbonyl oxygen of G305 [12]. Based on the structural studies, it is reasonable to assume that the p.S307C mutation, which is beside G306 and G305, might disturb the formation of the hydrogen bonds between UQ and G306/G305. Further in vivo studies are still needed to confirm the pathogenicity of S307C mutation.

## Conclusion

In summary, we described the phenotype and genotype of a late-onset GA II patient. Clinicians should be alert to young patients with unexplained muscle weakness and high CK levels. *ETFDH* gene screening is necessary for establishing the diagnosis and genetic counseling if muscle biopsy showed lipid accumulation.

## Additional files

Additional file 1: Figure S1. Histological findings of a control case. (TIFF 16416 kb)

Additional file 2: Table S1. Summary of three GA II patients with compound heterozygous mutations including p.S307C. (DOCX 20 kb)

## Abbreviations

ACMG: American College of Medical Genetics and Genomics
ALT: Alanine aminotransferase
AST: Aspartate aminotransferase
CK: Creatine kinase
CKMB: Creatine kinase-MB
EEG: Electrocardiogram
ETF: Electron transfer flavoprotein
ETF:QO: Electron transfer flavoprotein: ubiqionone oxidoreductase
ETFDH: Electron transfer flavoprotein dehydrogenase
GA II: Glutaric aciduria type II
HGMD: the Human Gene Mutation Database
LDH: Lactate dehydrogenase
MADD: Multiple Acyl-CoA dehydrogenase deficiency
MMT: Manual muscle testing
MRI: Magnetic resonance imaging
NADH: Nicotinamide adenine dinucleotide
PAS: Periodic acid-schiff
