# Supplementary figures and images for: Compound heterozygous mutations in electron transfer flavoprotein dehydrogenase identified in a young Chinese woman with late-onset glutaric aciduria type II
Source: Lipids Health Dis. 2017 Sep 26;16:185. doi: 10.1186/s12944-017-0576-5 (PMC5615764; doi:10.1186/s12944-017-0576-5)

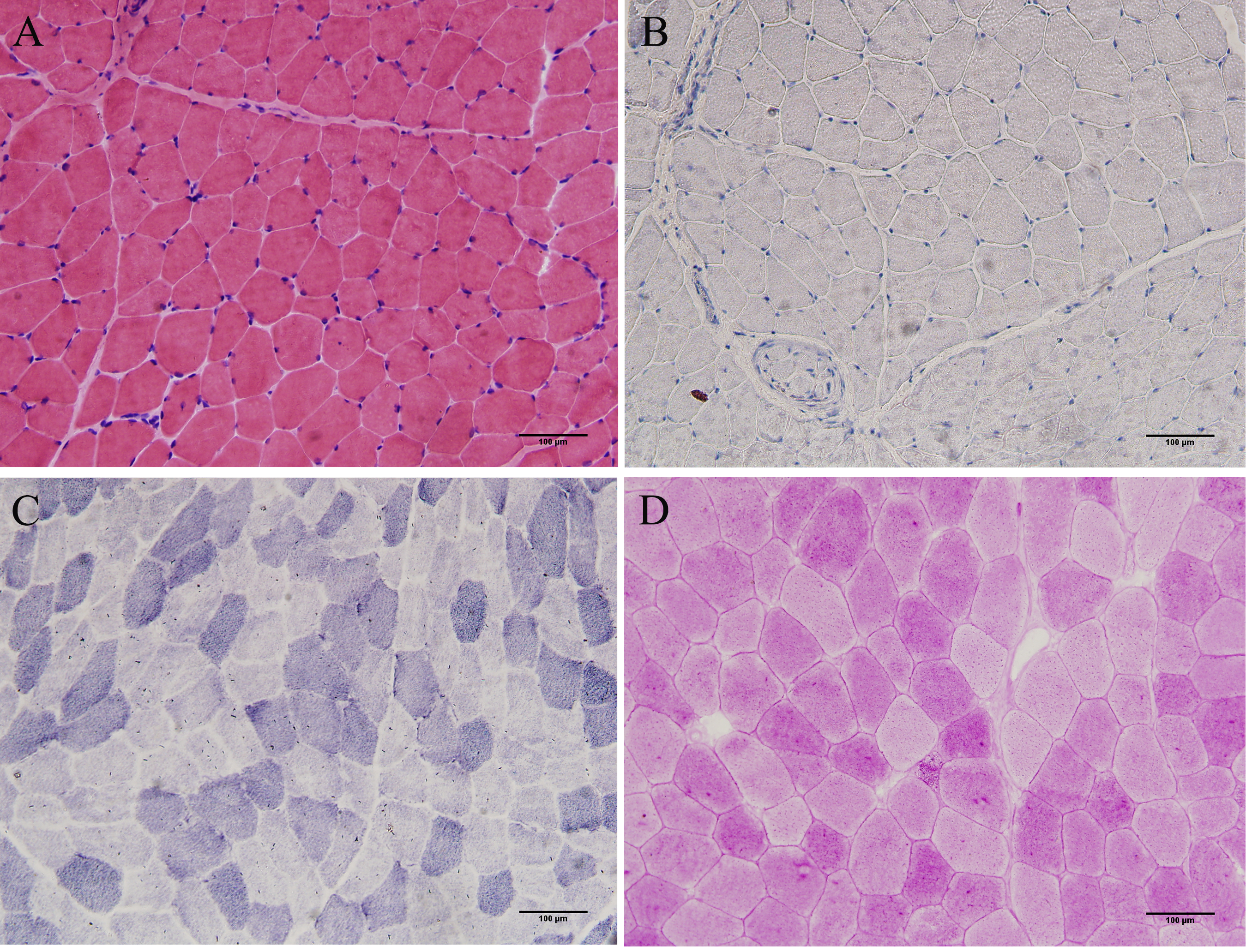

Supplement: Supplementary file 1 — Histological findings of a control case. (TIFF 16416 kb) [file 12944_2017_576_MOESM1_ESM.tif]
