# Supplementary material for: Compound heterozygous mutations in electron transfer flavoprotein dehydrogenase identified in a young Chinese woman with late-onset glutaric aciduria type II
Source: Lipids Health Dis. 2017 Sep 26;16:185. doi: 10.1186/s12944-017-0576-5 (PMC5615764; doi:10.1186/s12944-017-0576-5)
Supplement: Supplementary file 2 — Summary of three GA II patients with compound heterozygous mutations including p.S307C. (DOCX 20 kb) [file 12944_2017_576_MOESM2_ESM.docx]

**Supplement Table 1. Summary of three GA II patients with compound heterozygous mutations including p.S307C**

|  | **Patient1[**[**1**](#_ENREF_1)**]** | **patient2[**[**2**](#_ENREF_2)**]** | **Current case** |
| --- | --- | --- | --- |
| **Gender** | male | male | female |
| **Age** | 46 | 54 | 23 |
| **Mutation** | c.770A>G (p.T257C) | c.770A>G (p.Y257C) | c.250G>A (p.A84T) |
|  | c.920C>G(p.S307C) | c.920C>G(p.S307C) | c.920C>G(p.S307C) |
| **Symptoms** | Bent spine syndrome, limbs and lips numbness, absent deep tendon reflex | proximal limbs and neck weakness, distal limb and lip numbness, absent deep tendon reflex | muscle weakness, absent deep tendon reflex |
| **Electromyography** | No sensory nerve action potential | No sensory nerve action potential | Normal sensory nerve action potential |

## References

1. Peng Y, Zhu M, Zheng J, Zhu Y, Li X, Wei C, Hong D: **Bent spine syndrome as an initial manifestation of late-onset multiple acyl-CoA dehydrogenase deficiency: a case report and literature review.** *BMC Neurol* 2015, **15:**114.

2. Wang Z, Hong D, Zhang W, Li W, Shi X, Zhao D, Yang X, Lv H, Yuan Y: **Severe sensory neuropathy in patients with adult-onset multiple acyl-CoA dehydrogenase deficiency.** *Neuromuscul Disord* 2016, **26:**170-175.
